# Pricing practices of football and basketball clubs in Italy

**DOI:** 10.1057/s41272-021-00330-1

**Published:** 2021-06-22

**Authors:** Andreas Hinterhuber, Sara Viberti

**Affiliations:** Department of Management, Ca’ Foscari University of Venice, Venice, Italy

**Keywords:** Revenue management, Pricing, Dynamic pricing, Ticket prices, Sports, Football, Implementation

## Abstract

Revenue management is widely adopted across industries—except in the sports industry. In the context of the present study, we contact every single football and soccer club belonging to the top league in Italy—one of the world’s most successful football nations—and obtain responses from close to half of all clubs. We find that one-third of all football clubs apply revenue management, far more than basketball clubs. Basketball clubs rely heavily on fixed pricing, whereas soccer clubs use variable pricing or revenue management. Study data also suggest that perceived barriers to revenue management include a fear of alienating customers or perceived high implementation costs; since experiences of revenue management implementation are overwhelmingly positive, this study suggests that the fears of revenue management implementation are overblown. This study advances research on the practical implementation of revenue management in non-traditional industries and provides an encouragement for executives working in other industries to implement revenue management.

## Introduction

Revenue management is widely applied across different industries (McMahon-Beattie et al. 2016)—except the sports industry. This is quite surprising, since the sports industry has many characteristics that are conducive to revenue management implementation. This study thus has the objective of describing the state-of-practice of ticket pricing strategy in the football and basketball industry in Italy—one of the world’s most successful football nations. To do this, via professional connections of one of the authors, we contact every single football and basketball club in Italy’s top division and obtain responses from about 50% of clubs, both in football and basketball. We find that revenue management is relatively widely adopted in football—a third of the clubs polled—but not in basketball. Fixed pricing is common in basketball, but not in football, where variable pricing and revenue management are the norm and where fixed prices is virtually absent. We also explore perceived barriers to revenue management implementation and find that perceptions of high implementation costs and fear of alienating customers act as barriers to implementation. Since—with sample size limits in mind—the actual experience with revenue management implementation is overwhelmingly positive, the data in this study thus seem to suggest that the fear of revenue management implementation is mistaken. In conclusion: This study thus advances research on the practical implementation of revenue management in non-traditional industries and provides an encouragement for executives working in other industries to implement revenue management.

## Literature review

Revenue management is a pricing approach designed to ensure that the right number of tickets are sold to the right customers at the right price at the right place and at the right time (Kimes and Wirtz 2003; Queenan et al. 2011). The key elements of revenue management are price differentiation and market segmentation to align offer configuration with customer willingness to pay.

Revenue management is widely adopted across industries, also in the entertainment industry, an industry where the quality of the experience influences customer willingness to pay. Different studies highlight the relevance of seating position as a determinant of experience quality (Estelami et al. 2019; Rajagopal 2011; Serway and Vuille 2012). Price differentiation via multiple ticket categories for different seating positions increases revenues substantially: a study examining price differentiation for Broadway theater tickets finds a revenue benefit of 5% (Leslie 2004), as do studies examining the benefits of price differentiation for pop music concerts (Courty and Pagliero 2012; Eckard and Smith 2012).

The sports industry has the distinctive characteristics that characterize industries where revenue management is already widespread (Von Martens and Hilbert 2011). We will specifically discuss one key requirement: large variations in customer willingness to pay. The sports industry is dominated by the secondary market, which exploits the industry’s fluctuating demand and charges consumers different prices depending on game attractiveness. This practice is one of the primary reasons clubs should implement revenue management: the secondary market will thrive when tickets in the primary market are not optimally priced.

Differences in customer willingness to pay can be exploited by variable pricing and by revenue management (McMahon-Beattie et al. 2016; Nagle and Müller 2017). Variable pricing requires the creation of different offers sold at different prices based on a segmentation variable (e.g., quality of the game). Revenue management requires combining the segmentation variable with another important element: time. The main difference between these two pricing strategies is that with revenue management, ticket prices change up until game day, whereas with segmented pricing, ticket prices vary between each game but do not change during the on-sale period.

Revenue management can lead to dramatic price fluctuations—with damaging effects on customer relationships (Yeoman 2019): price floors and price ceilings are revenue management mechanisms designed to keep these fluctuations in check. Price floors protect season pass holders against low price offers that a revenue management system may propose (Drayer et al. 2012). Price ceilings ensure affordability for games that would sell out at any price: sports clubs do not want premium games to be considered exclusive events that only high-income fans can afford. Even with price floors and ceilings, customers may perceive revenue management as discriminatory (McMahon-Beattie et al. 2016). Fairness considerations thus lead ticket sellers to purposefully under-price (Kahneman et al. 1986).

Taken together, findings from the literature points toward potential benefits from implementing revenue management in the entertainment industry. We will therefore review studies discussing the actual implementation of revenue management in the industry and then present the results of our own survey examining the practice of revenue management in the Italian sports industry.

## Revenue management in the sports and entertainment industry

American sports organizations were the first to study the secondary market to better understand customers’ behavior as game day approaches. In 2009, the San Francisco Giants, a Major League Baseball team, became the first sports team to apply revenue management (MarketingSherpa 2009). The team reported an increase in ticket sales revenues of 7% after the first year of application (Drayer et al. 2012).

In the early 1990s, Cinema Plinius in Milan was the first to apply revenue management in Italy’s entertainment industry. The cinema’s ticket prices vary from €4.50 to €8.50 depending on several factors: screening time, time of purchase, opinions expressed on social media, movie typology, and actors’ reputation, etc. (Redazione 2016). Five weeks after introducing this new strategy, Cinema Plinius saw an increase in the number of spectators (+ 25%), in-person sales (+ 13%), and online sales (+ 110%) (Quarato, 2016). Other companies in the entertainment industry in Italy subsequently implemented revenue management with comparable results (see Table 1). The reported revenue improvements are considerably larger than the reported benefits of revenue management implementation in airlines of 3–7% (Skugge 2004).

**Table 1:** Revenue improvement—impact on company performance (source: Quarato, 2016)

Firm	Revenue improvement (%)	No. of spectators (%)
Plinius cinema	13	25
Filodrammatici theater	17	19
Zoomarine	15	15

The first sports organization to adopt the revenue management strategy in Italy was a football team: Virtus Entella. In January 2017, the sports team announced its new pricing policy in conjunction with Dynamitick, (Redazione 2017). Virtus Entella’s strategy is to charge less for tickets during the early on-sale period and then to increase ticket prices as game day nears. The idea behind this approach is to incentivize fans to purchase tickets in advance, enabling the team to profit from those fans who prefer to wait and make last-minute decisions. The algorithm Dynamitick developed to determine the optimal price for Virtus Entella’s tickets considers several factors, including the host team, weather forecasts, time of purchase, and more. The results at the end of the 2017 season were encouraging: revenues from ticket sales increased by 1%, and the number of fans increased by 3.54% (Quarato, 2016). A Dynamitick analysis demonstrated the positive influence of the revenue management strategy, with spectators confirming that the lower ticket prices encouraged auxiliary purchases, such as merchandise, food and drinks, and parking (Quarato, 2016).

Revenue management thus has the potential to increase firm performance as well as customer satisfaction. How widely diffused are revenue management practices in the European sports industry? Surprisingly, we do not have an answer to this important question. Objective of this study is thus an empirical analysis of the implementation of revenue management in Italy, a football-crazy nation with a national team that is still regarded as one of the strongest teams worldwide.

## Objective of our analysis and research methods

In this section, we analyze the pricing strategies adopted by Italian football and basketball teams. The objective of this analysis is to understand what considerations guide sports teams in determining their pricing policies. To obtain data for our analysis, we conducted a survey of all 17 “Serie A” basketball clubs and all 20 “Serie A” football clubs between February and June 2020. The survey focuses on the elements the sports clubs consider when implementing their pricing policies, the ticketing strategies they adopted (fixed pricing, variable pricing, and revenue management), and the reasons behind their choices. Overall, 11 basketball clubs and 9 football clubs replied to the survey, representing an overall response of 54% (65% for the basketball market and 45% for the football market). The basketball clubs surveyed represent 65% of the Italian basketball market’s total seating capacity. The football clubs represent 42% of the Italian football market’s seating capacity. This survey is thus a relatively comprehensive survey of pricing practices in football and basketball tickets in Italy.

The 2019–2020 season was interrupted in March because of the COVID-19 pandemic; however, this early termination does not affect our analysis since the pricing strategies were decided and developed beforehand. Details about the professionals who replied to our survey are provided in Table 2.

**Table 2:** Respondents

Sport	Job title	Data collection method
Basketball	Marketing Manager	Online survey
	Marketing and events Manager	
	Marketing Manager	
	Communication and Social Media Manager	
	Ticketing Operations	
	Ticketing Manager	
	Marketing and Sales Account	
	Marketing and Administration Manager	
	Ticketing Manager	
	Sales and Marketing Manager	Telephone Interview (25 min)
	Marketing Officer	Telephone Interview (20 min)
Football	Chief Revenue Officer	Online Survey
	Corporate Hospitality and Ticketing Manager	
	Ticketing Premium Coordinator	
	Ticketing Coordinator	
	Ticketing Manager	
	Sales Manager	
	Marketing Project Manager	
	Sales Manager	
	Operations Manager	

We begin our analysis by exploring the factors the sport clubs considered when determining their prices. We identify three broad groups of pricing practices: fixed pricing: prices are uniform and price segmentation occurs only via seating position and age-based discounts; variable pricing: prices vary depending on one or more segmentation variables reflecting willingness to pay: quality of the host team, weather forecast, day of the week, proximity to festivities, and others; revenue management: prices vary depending on segmentation variables (as above) and time of purchase: price fluctuates in response to demand changes during the on-sale period.

Revenue management represents the most developed and innovative strategy as it attempts to maximize revenue and attendance at the same time. Table 3 synthesizes the elements considered by the basketball and football clubs that answered the survey.

**Table 3:** Pricing strategies applied

	Fixed pricing	Variable pricing (Host team variable)	Variable pricing (Time of the year and the day of the week variables)	Dynamic pricing	Total
No. of Basketball clubs	4	2	4	1	11
Percentage	36%	18%	36%	9%	100%
No. of Football clubs	0	5	1	3	9
Percentage	0%	56%	11%	33%	100%
Total	4	7	5	4	20
Percentage	20%	35%	25%	20%	100%

**Fig. 1:**
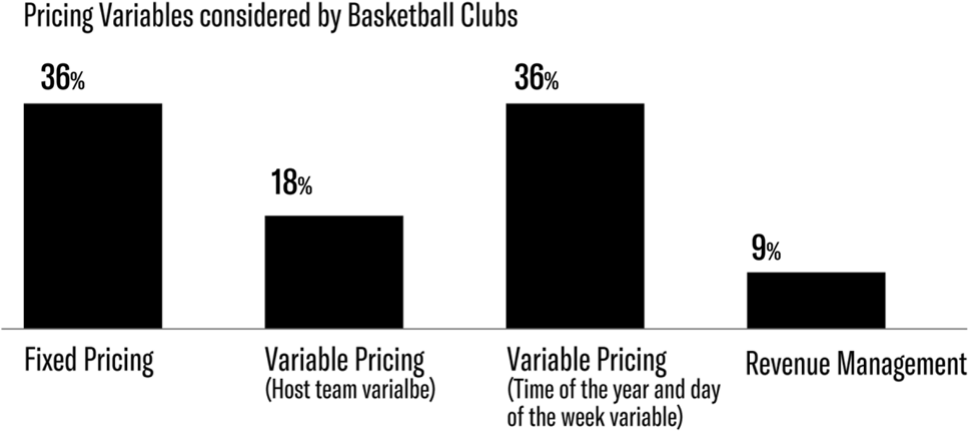
Strategies applied by the sport clubs analyzed

**Fig. 2:**
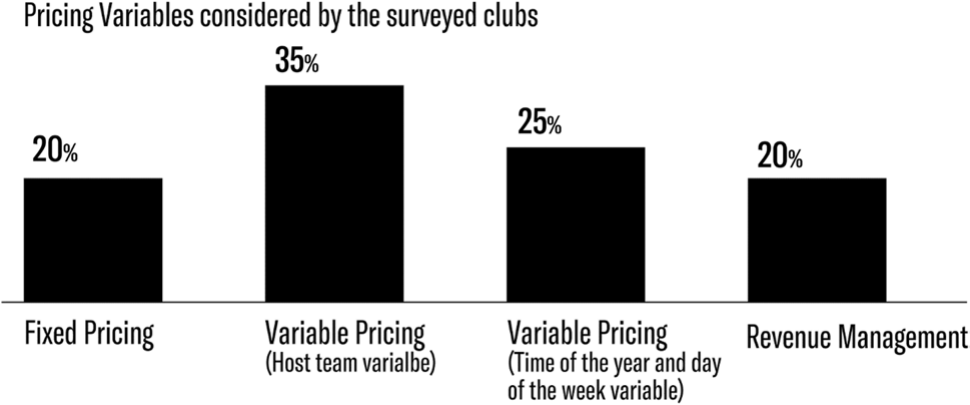
Strategies applied by the basketball clubs analyzed

Table 3 and Figs. 1 and 2 highlight the approaches football and basketball clubs follow in their pricing policies. Variable pricing comes in two shapes: variable pricing based on host team quality and variable pricing based on host team quality and game time. Revenue management, finally, considers several variables and adjusts ticket prices according to game demand.

From Table 3 and Fig. 2, it is clear that the basketball market considers revenue management as too sophisticated since only one team adopts this strategy. All of the basketball clubs analyzed adjust their prices based on the different experiences offered by the distinct seating options. Most of them adopt discounted fares to attract certain groups of customers, especially children. The basketball clubs that decided to adopt the fixed pricing strategy are guided by many different reasons. Some chose this pricing strategy because season tickets account for a high percentage of total seats sold: the potential benefits of revenue management are clearly more limited for clubs that sell a large share of tickets in advance.

The majority of basketball clubs implement variable pricing. As shown in Fig. 2, this strategy considers two variables: host team quality and the time of the game. Surprisingly, only six out of the 11 surveyed basketball teams consider host team quality, an element that affects game demand, and only four out of these six consider other elements (e.g., game time) as well. Moreover, 18% of the basketball clubs that initially analyzed host team quality decided not to include this variable in their pricing policies. Only one basketball club applies revenue management: variables considered include the host team quality, game attractiveness, day of the week, game time, time of the year, and concurrence with other events. Prices vary substantially all the way up to game time.

**Fig. 3:**
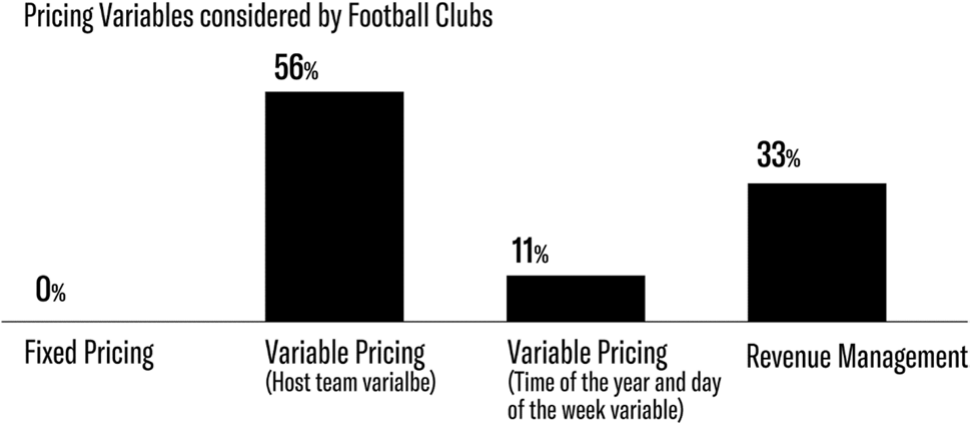
Strategies applied by the football clubs analyzed

Pricing practices adopted by football teams are, on average, far more sophisticated. Our analysis, based on data from 9 out of 20 “Serie A” teams, provides the following insights: As Table 3 and Fig. 3 show, revenue management is quite common in football, with three out of the nine teams adopting revenue management. Interestingly, none of the football teams applies fixed pricing: football clubs thus seem to be highly aware of the importance of identifying factors that influence customer willingness to pay. Of the teams analyzed, 56% implement variable pricing that considers host team quality and reputation, and only 11%—one club—include game time as well.

As indicated, 33% of the teams implement revenue management. In practice, the teams divide the on-sale period into different intervals and choose the price ranges applied in each interval. Therefore, prices do not fluctuate freely during the on-sale period. Clubs apply this strategy because it allows teams to analyze different variables and to exploit customers’ willingness to pay by learning from the experience of prior periods. The variables that are considered include host team quality, historic attendance of similar games, partnerships with the host team, day of the week, weather forecast, and available TV coverage.

## Discussion

Italy is one of the leading football nations globally. We had unique access to marketing managers of about half of the football clubs in the top league in addition to managers of over half of the basketball clubs in the top league.

Overall, in our analysis, we find that revenue management is not yet broadly adopted, but more common for football than for basketball clubs. The reason few clubs adopt this strategy is that many find it too difficult to develop and manage the pricing fluctuations during the on-sale period.

As a result, most of the surveyed sports clubs decided that the variable pricing strategy or the fixed pricing strategy suited their needs better than revenue management. Some clubs adopted fixed pricing to attract as many spectators as possible due to low team performance, while other clubs decided to focus on season ticketholders, setting fixed prices for the remaining seats. Most teams, however, prefer the variable pricing approach as it limits price fluctuation to known, expected factors as opposed to including price fluctuations induced by demand peaks. With variable pricing, price does not change in response to fluctuations in actual demand; rather, price changes are determined according to forecasted demand. However, clubs that utilize variable pricing find it more difficult to adjust their prices in response to unexpected shifts in demand because this strategy remains fixed to some degree. Thus, the rigidity of the more common variable pricing approach prevents some of the positive effects that revenue management would bring to clubs.

Clubs find it challenging to engage in revenue management because of their fear that customers retention and future purchases may suffer at the expense of current prices and profits (Yeoman 2019). Some teams argue that the market is not ready for revenue management yet.

In comparing the two markets explored in our study, the basketball market appears to prefer variable pricing as many clubs consider revenue management too sophisticated for their teams and fans even though the strategy may lead to increased revenues. The football market is more advanced in this regard because more clubs implement revenue management, and no team has a fixed price policy.

Finally, our research finds that segmentation variables for variable pricing differ between football and basketball. In both cases we observe age-based price discrimination (reductions for youngsters and elders), but only the football clubs in our sample offer discounted tickets to female customers. The basketball fan base already comprises a strong percentage of women, while football fans are primarily men. Price discrimination in football clubs thus aims at attracting an under-represented customer segment that has, presumably, a lower willingness to pay.

## Conclusions

Revenue management is coming of age. After the San Francisco Giants’ case and other American sports organizations’ experiences, Italian sports organizations began applying the strategy as well, starting with Virtus Calcio Entella (football) and Auxilium Pallacanestro Torino (basketball). This study is the first comprehensive empirical survey of pricing practices in Italy’s top football and basketball leagues. Our study offers some fascinating insights.

Our analysis reveals that variable pricing remains the most widespread strategy applied by Italian basketball and football clubs, with football clubs implementing revenue management (33%) far more than basketball clubs (9%). Many clubs are still skeptical about the opportunities offered by revenue management. Their main concern is about fans’ perceptions of price: if price changes are perceived as unfair, customers are less inclined to repeat their purchases. Our study reveals that the reluctance that clubs show vis-à-vis revenue management is, in fact, unfounded: The positive results obtained by the clubs that apply this pricing strategy show that customers are already comfortable with price fluctuations over time, quite likely resulting from positive experience with other services that do not have static prices (e.g., airlines, hotels).

Our analysis also reveals two completely different markets in terms of pricing and attendance. Basketball has loyal fans and a high share of season tickets, football has a declining number of loyal spectators. Thus, in basketball, the objective of pricing is keeping spectator loyalty high. Not so in football. The objective of pricing and, specifically, of revenue management is to increase attendance and profits. Revenue management clearly cannot achieve this in its own—a strong, differentiated customer experience is a pre-requisite—but revenue management clearly creates a dialog between football teams and fans and is a powerful instrument to align demand with capacity via pricing.
